# Vitality and Its Co-ordinate Forces

**Published:** 1873-10

**Authors:** W. T. Grant

**Affiliations:** Georgia


### VITALITY AND ITS CO-ORDINATE FORCES.

BY W. T. GRANT, M. D., OF GEORGIA.

The thoughts and suggestions presented in the following essay are remarkable for novelty, at least, if they possess no other excellence. They are offered with the hope that they will stimulate thought and research in this direction. To those who may be disposed to regard them as wild and visionary dreams, I have only to say, that when they shall have investigated these subjects fully and thoroughly, they will have the right to express an opinion on them, but not before. And I would suggest to every one who may read this essay, that they should study it well and be sure that they understand it in all its bearings, before they shall make a final decision.

A plant growing in the open air, freely exposed to the sunlight and the rains and dews, is in that situation best adapted for health and growth. The rootlets in the ground absorb the rains loaded with all the soluble elements of the air and soil, and convey into the body of the plant all the *material* elements necessary for its future growth. I except, of course, some gaseous elements which enter directly from the air, through the green parts of the plant. Here we have what is believed to be the whole material or matter which contributes to the growth of the plant. I do not think that this is all that enters into its composition. While the plant is growing, the different *immaterial* elements which compose the rays of the sun are deposited in the tissues of the plant, and enter into the composition of vegetable parenchyma, as truly as the carbon or potash or any other element. These *immaterial* elements of the sun's rays are called the thermal, the luminous and the chemical or photogenetic rays. To these may be added a fourth element, not derived from the sun's ray, but which is universal, and this is electricity. These four immaterial elements always enter into the composition of every vegetable fabric, and constitute an integral part thereof.

The thermal and luminous rays, or elements, as I prefer to call them, are again given out or escape from their late combinations when we burn wood of any kind, and become again manifest in the heat and light then emitted.

Hence, all the heat and light which arises from burning vegetable matter, is only the same heat and light deposited there during the growth of the plant. If this be not so, then every act of burning and every chemical operation is an act of creation, creating light and heat. Will any one maintain such a proposition ?

Intimately connected with this subject is that of latent heat. It is customary to call all heat that disappears in natural operations, that is, when it ceases to affect the thermometer, *latent heat.* In the change of any substance from a solid to a fluid condition, a certain amount of heat thus disappears, as occurs in the change of ice into water. This heat will again reappear when the water is frozen. In accordance with the theory above, this heat should be regarded as a chemical element, combining with the ice, and the compound resulting being water. I will explain this further. Ice is a solid. When a certain amount of heat has been brought in contact with it, the heat combines with it chemically, and as a consequence, its nature is changed ; it is no longer ice, but water. And if a further increment of heat be added, the water will cease to be water, and become a vapor which we call steam. In each of these cases, nothing has been done but to supply heat, which disappears entirely. I can see no difference between this operation and that which would occur if oxygen were brought in contact with potassium ; the heat and the ice both disappear in the one case, producing a new body-water, and the oxygen and potassium disappear in the other, producing similarly a new body-potash. The oxygen can be made to reappear ; so, also, can the heat. And if the one is a true chemical operation, I cannot see why the other should not be the same. Hence, I hold that the operations of heat are purely chemical. I also claim the same for electricity, light and the chemical ray of the sun light.

I will now suggest another thought collateral to all the above. A sun's ray, commonly called a ray of white light, is composed, as already stated above, of three or four elements—of light, heat and a chemical ray. These elements are supposed to be simply in juxtaposition, (why not in combination ?), and thus together constitute the sun's ray. Sir Isaac Newton, with a prism, separated the light from the other elements, and showed that it was a compound made up of the several different colors exhibited in nature. I now ask the question, may not the other elements of the sun's ray also be compounds, and is it not possible to decompose them likewise, as Newton did the light ? Who shall be the fortunate person to do this ? It seems to me that within the compass of this suggestion, lies the explanation of a vast deal of both animal and vegetable physiology.

It is almost a trueism in Philosophy, that light, heat, electricity, and the chemical force are mutually convertible, or that this is true at least of the two former, and I believe it equally true of the last. I wish the reader to bear this strictly in mind in what follows, as I now enter upon the most important part of this monograph.

Prof. Darwin uses the following language: "In what manner the mental powers were first developed in the lowest organism, is as hopeless an inquiry, as how life first originated." If what follows on this and kindred subjects is not wholly visionary, we may be able to account for both life and the mental powers. And I ask a careful study of the following exposition:

Man derives his entire nourishment *ultimately* from the vegetable kingdom, either directly as when he eats vegetable products, or secondarily when he eats the flesh of animals which have lived upon vegetables. Indeed, the entire animal kingdom receives its support from plants, without exception.

I take the position that the different vital manifestations, as life, mind, muscular power, &c., are but the equivalents of the light, heat, electricity and chemical force, which disappeared during the growth of the plant, and now re-appear by conversion in these forms. Or, to be more explicit, mind, vitality, and the other animal forces, are but other and new forms of the heat, light, electricity and chemical force which the vegetable organism absorbed during its growth directly from the sun; and, are, therefore, the same thing in other forms. These immaterialities are introduced into the system in the food, and there, by conversion, become the different vital forces.

I may perhaps better explain my meaning by an illustration. There are an immense number of small bodies in space, which appear to revolve around the sun in an orbit, whose plane cuts the plane of the earth's orbit with a considerable angle. And in consequence of this, when the earth reaches those parts of its orbit which are cut by the orbit ot these small opaque bodies, it must necessarily meet some of them, and they fall upon its surface, constituting the well known meteoric stones. When they first strike the earth's surface they are intensely hot. This circumstance was, for a long time, a great trouble to philosophers to explain. Where did the heat come from? It is now explained as follows: The meteorite in its passage through space, possessed a great velocity, which involved great force. When it strikes upon the earth, this motion and force, being thereby suddenly stopped, are converted into heat, as nature never suffers anything to be lost, whether it be material or immaterial. The force, in this case, instead of being destroyed or lost, is converted into heat. Another illustration of the same thing, is seen in the heat produced by hammering upon anything. The heat of friction is another instance. They are only force converted into heat.

I presume that I am now understood in saying that all the normal forces of the body, as vitality, mind, muscular power, &c., have their origin in these immaterial elements of the food, and are simply the equivalents of so much heat, electricity, and chemical affinity, originally deposited or chemically combined in the vegetable structure.

In like manner the abnormal forces of the body, as pain, fever, &c., have the same origin. Some diseased action perverts the healthy converson of these elements, and they are changed into pain, fever, &c., instead.

In the same way we may account for insanity, and the various exhibitions we have of perverted inervations, as manifested in epilepsy, catalepsy, ecstacy, &c.

If we believe that these forces, or, as I am disposed to term them, elements, are the active motor powers in this department of nature, then the thought occurs that here is certainly a new division of chemistry, which may be called mental or metaphysical chemistry, which deals only with the immaterialities of nature. And we see that these immaterialities are as important integers in chemistry as the most tangible of those elements to which it has heretofore confined itself.

If these views should ever be accepted, many other important thoughts will be developed as sequences, which without any figure of speech, are too numerous to mention.

It will be seen that I have attributed to light, heat, the chemicals force and electricity, the origination of all the animal or vital forces by conversion. And I trace these, or at least the three first, through the food to the vegetable kingdom, in which they were deposited during the growth of the plants, by the sun. Hence our grand conclusion—all the vitality on the earth is derived from the sun, or in other words, *the sun is the source of all life and being.* And we may go a step further, and approach another great question, from a new and unexpected direction. If the sun has given life to our earth by means of the light, heat and chemical force of its rays, it is but reasonable to believe the same of the other planets. And we may believe that life in some form exists wherever in the vast universe such light as ours is to be found.

With that wonderful instrument, the spectroscope, philosophers have taken light in hand and made it tell the story of the material and chemical composition of the sun and other worlds. May it not be possible to make the same element, or one of the others, disclose to us the complimentary tale that life and its co-ordinate forces are also there?

